# Non-clinicians’ judgments about asylum seekers’ mental health: how do legal representatives of asylum seekers decide when to request medico-legal reports?

**DOI:** 10.3402/ejpt.v3i0.18406

**Published:** 2012-10-16

**Authors:** Lucy Wilson-Shaw, Nancy Pistrang, Jane Herlihy

**Affiliations:** 1 Camden and Islington NHS Foundation Trust; 2 Division of Psychology and Language Sciences, Faculty of Brain Sciences, University College London, UK; 3 Centre for the Study of Emotion and Law, London, UK

**Keywords:** Refugees, asylum, decision-making, psychological assessment, vulnerability assessment

## Abstract

**Background:**

Procedures for determining refugee status across Europe are being speeded up, despite the high prevalence of mental health difficulties among asylum seekers. An assurance given is that ‘‘vulnerable applicants’’ will be identified and excluded from accelerated procedures. Although experts have recommended assessments to be undertaken by experienced clinicians, this is unlikely to happen for political and financial reasons. Understanding how non-clinically qualified personnel perform assessments of mental health issues is timely and crucial. Misrecognition of refugees due to the inappropriate use of accelerated procedures involves the risk of returning the very people who have the right to protection from further persecution.

**Objective:**

To examine the decision making of immigration lawyers, who are an example of a group of nonclinicians who decide when and whether to refer asylum-seekers for psychiatric assessment.

**Method:**

Semi-structured interviews were conducted with 12 legal representatives working with people seeking refugee or human rights protection in the United Kingdom. The resultant material was analysed using Framework Analysis.

**Results:**

Themes clustered around the legal case, the client, the representative and the systems, all with sub-themes. A mapping exercise integrated these themes to show how representatives brought together questions of (1) evidential reasons for a report, influenced by their legal, psychological and case law knowledge, and (2) perceived evidence of mental distress, influenced by professional and personal experiences and expectations.

**Conclusions:**

The legal representatives interviewed were well-informed and trained in psychological issues as well as clearly dedicated to their clients. This helped them to attempt quasi-diagnoses of common mental health problems. They nonetheless demonstrated stereotypical understanding of post-traumatic stress disorder and other possible diagnoses and the role of subjectivity. The study has implications for other groups – particularly those less trained and compassionate – who are required to make clinical judgments without the necessary expertise.

A refugee is defined in the United Nations’ *Convention relating to the Status of Refugees* as a person who has a “well-founded fear of being persecuted for reasons of race, religion, nationality, membership of a particular social group or political opinion”, who is outside of his or her country of nationality and who is “unable or unwilling” to return to the protection of that country (United Nations, 1951). All signatories to this international agreement (including all European states) are committed to providing protection to people arriving in their country who fit this definition^1^, but the precise procedure for determining status is left to each state to define^2^. This usually involves the claimants giving an account of persecution in their home country and an explanation of why this leads to their having a “well-founded fear” of return. Given the difficulty of most applicants in providing documentary evidence, witnesses or other corroborating evidence, the final decision usually rests on what is known about the country in question and on the credibility of the individual applicant.

In the 1990s, these decisions often took many months, sometimes years (Good, 2004). Partly in response to this, many European countries have introduced accelerated procedures. For example, in the United Kingdom, timeline targets were introduced in 1999 and the Detained Fast Track (DFT) formally came into being in 2003.

It is UK Border Agency policy that any asylum claim, whatever the nationality or country of origin of the claimant, may be considered suitable for DFT … [Detained Fast Track] processes where it appears, after screening (and absent [sic] of suitability exclusion factors), to be one where a quick decision may be made. (Home Office, 2012b, p. 3)

`

In practice, once someone has been interviewed in accordance with the DFT procedure, their claim is decided within 2 or 3 days. If refused (96% of claims were refused on first instance in 2008), they have 2 working days to make an appeal, which must be heard within 11 days (91% were denied in 2008). They are kept in detention throughout this process and until eventual removal from the country (Home Office, 2012b, p. 3; Human Rights Watch, 2010).

These procedures are a matter of concern to clinicians who work with people seeking asylum, who are aware of the high rates of psychological distress in this group, and the associated barriers to the prompt disclosure of traumatic experiences.

A recent enquiry into the DFT in the UK reported that

Of 114 cases sampled, 30% were taken out of detention at some stage and 27% of these were released before a decision on their asylum claim had been made. Most of these people (44%) were released due to health issues and evidence they were victims of torture or trafficking. (Vine, 2011, p. 3)

However, they also concluded that

While safeguards were in place once people had been detained, there remained a particular risk that the victims of torture or trafficking could be allocated to the DFT contrary to the Agency's own policy. (Vine, 2011, p. 3)

## Psychologically informed understanding of claims

Rates of psychiatric diagnosis amongst people seeking asylum are known to be high (Cembrowitz & Burnett, 2003; Fazel, Wheeler & Danesh, 2005; Ouimet, Munoz, Narsiah, Rambure & Correa, 2008), so there is reason for concern about the welfare of those within such accelerated procedures.

However, this is not just a question of the welfare of applicants. In terms of presenting a case for state protection, there are further reasons as to why mental health difficulties must be recognised and documented. To be deemed credible, asylum applicants need to present a coherent, consistent and prompt account of their claim (UK Border Agency, 2011). The ability to consistently recall details of traumatic experiences over repeated interviews has been shown to be impaired by post-traumatic stress disorder (PTSD) (Herlihy, Scragg & Turner, 2002). Similarly, delays in disclosure of distressing personal experiences, including sexual violence, are associated with higher PTSD symptom levels as well as PTSD avoidance symptoms, dissociation at the time of interview and shame (Bogner, Herlihy & Brewin, 2007). Showing that these psychological difficulties pertain to the one who is applying to be recognised as a refugee could be crucial to the decision makers’ understanding of the person appearing before them. Failure to understand these issues could mean a decision to return someone to further persecution and even death.

State procedures for claiming asylum include assurances that “vulnerable” applicants are excluded from such “fast-track” procedures at an early stage. For example, in the Netherlands, the “general asylum procedure” takes 8 days, but there is provision for an “extended asylum procedure” in case of requiring, among other reasons, medical research.

The United Kingdom has similar procedures (Home Office, 2012a). The United Nation High Commission for Refugees (UNHCR) carried out an audit of the implementation of such policies concluding that

UNHCR considers it imperative that where factors come to light which render it possible that a claim may not be decided quickly, Case Owners should proactively consider the removal of the claim from the DFT, irrespective of whether an express request for removal has been made. Training should be provided on the identification of particularly vulnerable claimants who may be unable to present their claim in the short timescales available in the DFT. (United Nations High Commission for Refugees, March, 2008, p. 22, section 2.3.78)

## Vulnerability assessment

This raises the question of assessment of vulnerability that takes place. Who performs these assessments, what is their definition of “vulnerability” and on what observations or other evidence do they base their judgment?

Current “vulnerability assessment” in the United Kingdom is made largely on the basis of group membership. People excluded from the “DFT” include women who are 24 or more weeks pregnant; unaccompanied asylum-seeking children; those with a medical condition requiring 24-hour nursing or medical intervention; those who have evidence of trafficking from a “Competent Authority”; and those with “independent evidence” of torture (Home Office, 2012b, section 2.3).

The last of these criteria is problematic as it rests on an issue which is also subject to credibility assessment. If immigration officials were to remove from the normal process everyone who *alleges* experience of torture, it would clearly invite false claims. On the contrary, independent evidence, immediately on arrival in a host country, can be hard to come by. These categories also do not recognise the role of mental health problems in presenting a credible claim for asylum regardless of the nature of the substantive claim for asylum.

Research has shown that recognition of mental health problems outside of the specialist mental health professions is often poor. Studies have found that only around 50% of patients who meet the criteria for a psychiatric diagnosis are recognised as having psychological problems by their doctor (General Practioner) (Lecrubier, 1998; Ormel, Koeter, Vandenbrink & Vandewillige, 1991), and the recognition rate for PTSD is even lower (Leibschutz et al., 2007).

Whether they be for the identification of “vulnerable applicants” or a full explanation of the psychological issues in an individual's claim, there has been no study on how mental health assessments are done, de facto, by the non-clinical personnel involved in the asylum process.

## UK legal representatives

Whilst in theory, anyone who comes into contact with a person seeking sanctuary in his or her country might recognise that the applicant needs psychological help, in practice, apart from immigration officers, the person's legal representative is the one most likely to identify a problem. Legal representatives are in a position to recognise the ways in which an applicant's mental health may be a cause for concern – either in terms of their care or the preparation of their claim. Also, it is by claimants – usually through instructions issued by their legal representatives – that medico-legal reports are usually requested to fully assess and explain any apparent psychological difficulties. Medico-legal reports are not requested for all legally represented clients – indeed, restrictions in the public funds available to asylum seekers has meant that funds are less easily obtainable for expert reports. However, it is one of the avenues of evidence-gathering available to lawyers and, where they are commissioned, it is invariably by a legal representative.

## Objective

Immigration lawyers’ decisions to refer clients for medico-legal reports provide an opportunity to study non-clinically trained professionals undertaking what is essentially a clinical assessment in itself. We have found no published literature exploring how legal representatives recognise mental health problems or how they decide when to refer for a psychological medico-legal report. Our objective therefore was to understand how legal representatives decide when to commission psychological reports in asylum cases. A qualitative approach was adopted, as it is well-suited to exploring complex processes (Willig, 2008). Through a systematic, in-depth analysis of a small number of accounts, we aimed to gain an understanding of the decision-making process.

## Method

### Sample

The study was advertised on an extensive email group of UK legal representatives (708 members). Eleven members currently involved in asylum case work agreed to take part. One more person was recruited through a direct call to a law firm. Of the ten women and two men participants, nine were solicitors, one a barrister and two were case workers. All the participants had 6 or more years’ experience of working with asylum cases, with a range of 6–27 years and a mean of 9.87 years. All were actively involved in asylum casework in a legal environment.

### Procedure

Information about the study was sent to all participants by e-mail and interviews were arranged at their offices. One day before the interview, participants were sent an e-mail explaining the structure of the interview and asking them to select three cases – one where a medico-legal report had been requested, one where it had not been requested and one where it had been considered but eventually not requested. During the interview, participants had a further chance to read the study information and give written consent.

### Interview

A semi-structured interview format was used. The interview began with an open question about what factors the participant considered when thinking about whether to request a psychological medico-legal report in a case. The researcher then followed up on the factors raised by the participant to get a more in-depth understanding. The second part of the interview asked the participant to talk through their decision-making in the three pre-selected cases. Follow-up questions explored how they perceived their client had felt about the case, the participant's expectations for the report, the usefulness of the report, the outcome of the case and, if the report made recommendations, whether these were followed. Interviews lasted approximately 1 hour. All interviews were recorded on a digital voice recorder and transcribed by the first author verbatim.

### Analysis

The interview transcripts were analysed thematically, using the National Centre for Social Research “Framework” approach (Pope, Ziebland & Mays, 2000; Ritchie, Spencer & O'Connor, 2003). This approach, developed for use in social policy research, aims to understand complex behaviours and systems and is useful for answering questions such as “why are decisions or actions taken, or not taken?” (Ritchie & Spencer, 1994, p. 174). Following the recommended steps (Ritchie et al., 2003), a thematic framework was constructed and systematically applied to all the transcripts; thematic charts were then produced for each theme, documenting the supporting data from each interview. A final “mapping” stage examined links between themes with a view to synthesising the material into a parsimonious, descriptive and explanatory account.

Guidelines for good practice in qualitative research were followed (Elliott, Fischer & Rennie, 1999; Mays & Pope, 2000; Pope & Mays, 2006). A consensus approach was used at each stage of the analysis; the development and application of the thematic framework were discussed by members of the research team, and the analysis of four randomly selected transcripts was audited by a second researcher. Respondent validation was also carried out; each participant was sent a summary of the themes from their interview and asked to provide feedback. Eight of the twelve participants provided feedback; five agreed fully that the summary was an accurate reflection of their views and three made some minor corrections (such as adding qualifiers to statements) but generally agreed with the summary. Their feedback was incorporated into the analysis at the mapping stage.

### Ethics

University College London granted ethical approval for the study.

## Results

The analysis yielded four main themes, each with several sub-themes, concerning how decisions were made about when to request psychological medico-legal reports in asylum cases. The themes and sub-themes, along with examples of each, are shown in Table 1.

**Table 1:** Main and sub themes, with examples

Main theme	Sub-theme	Examples
Case factors	Available documentation	Existing medical evidence
		Recorded mental health history
		Reasons for refusal letter
	Reported events	Torture/detention
		Rape/trafficking
	Likely credibility	Inconsistencies
		Late disclosure
		Inappropriate emotional reaction
Client factors	Presentation	Signs of distress
		Symptoms of mental illness
		Suicidality
	Abilities	Inability to understand/answer questions
		Inability to cope with daily life
	Agreement with referral	Refusal (cultural taboo/stigma)
	Family circumstances	Supportive family
		Problems in family members
Legal representative factors	Knowledge, experience and personal style	Knowledge of case law
		Training on mental health
		Desire to help
		Confidence in own ability
	Networks	Links to specialist organisations
		Professional relationships
	Ethos of organisation	Provision of training
		Spending time and money
Systemic factors	Legal system	Applications for funding
		Applications for adjournments
		Evaluation of medical evidence
	Medical system	Access to services
		Waiting lists
		Reluctance to write reports

**Fig. 1:**
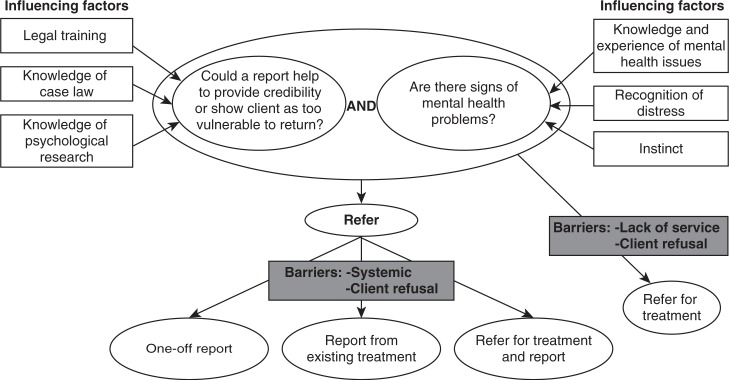
Mapping of the decision to refer a client for a medico-legal (psychological) report.

The “mapping” stage of the analysis integrated these themes to capture the process of decision-making. A visual representation of this is presented in Fig. 1.

### The decision to refer

Two main factors in conjunction seemed central in prompting a decision to refer for a psychological medico-legal report. The first was the judgment that a report would be helpful for the case; this was primarily related to issues with the credibility of the clients’ account and risk (to their mental health) on return.

Deciding whether it's necessary, whether it assists with the claim … Either you're using it for value to say that the experience is one that it's likely that the person has gone through. The other is to say, actually this person can't go back. (Participant 6)

This factor appeared to be influenced by the participants’ knowledge of case law regarding the use of medico-legal reports, their legal training and their knowledge of psychological research which could potentially be used to back the need for a report.

I have quite good knowledge about trauma and effects on credibility, inconsistencies, all of [Dr X's] research. (Participant 1)

The second factor was some indication that clients had a severe mental health problem. Participants identified different ways through which this could be brought to their attention, the clearest being if it was identified by existing documentation of the case; if clients were already in contact with mental health services, a report would automatically be requested.

The first thing is obviously whether they're getting any psychiatric or counselling treatment, and if they are then that's … a given that we'll have some sort of report. (Participant 7)

When clients were not already labelled as having a mental illness, participants said that they primarily relied on the presentation of the client. Signs of potential mental illness identified by participants were elevated levels of sadness, the client being ‘upset’, aggression or withdrawal.

[Interviewer: what kind of things would make you think this person is mentally distressed?] Oh, you know, if they're crying in appointments, if they can't talk about things or become obviously upset about talking about things. (Participant 7)

The way they're displaying in body language and in language, they're reluctant to talk about it, very tearful. (Participant 4)

As well as the importance of seeing signs of distress, there was also a sense of the need for this distress to be severe enough to warrant a diagnosis. Participants reported that they asked particular questions to clients to ascertain whether their presentation would merit a diagnosis; some of the questions seemed to come from knowledge and training on mental health issues and related to symptoms of PTSD and psychotic illnesses, whereas others seemed less directly related to mental illness. It was expressed that if these “symptoms” were not present, then there would be no benefit in referring for a report, as they would not gain a diagnosis.

He said he wasn't suffering from anything you would expect to see on a PTSD assessment. [Interviewer: What kind of things did you ask him about?] How do you sleep, do you have intrusive dreams, do you have nightmares, do you think about things during the day, do you remember things and do they upset you, do you feel sad, do you find it difficult to talk to people, do you not want to make friends. (Participant 7 – discussing a case not referred)

Cases where I wouldn't consider a report is [sic] where … they are not going to be considered acute enough to be able to access the services of a psychologist, psychiatrist, a mental health team. (Participant 2)

Depression was generally perceived as not being grave enough by itself to warrant referring for a report, as it was thought that courts would not give any weight to depression alone.

If he's inconsistent and you say, well look the poor man's depressed, they'll laugh you out of the court room. You're just not going to get anywhere. (Participant 1)

Some participants highlighted the fact that they could miss things, particularly when clients were not vocal in expressing their problems. In a case where a client was referred for a report during an unexpected lull in the case because he had experienced the same events as his wife (who had severe psychological problems), the legal representative was surprised by the report, which she had expected would find no psychological issues.

We got a psychiatric report and it said well actually this man's suffering from moderate to severe depressive episode, and he suffers from PTSD. Which had surprised me because he hadn't put forward particularly that he'd been affected by traumatic events (Participant 7)

The events which a client reported experiencing were seen to be a further indicator of mental illness. There appeared to be a view that if someone had experienced certain events, such as rape, trafficking or torture, then they “should” show psychological effects of this.

With the rape cases we always refer them straight away. (Participant 11)

You think well that can't possibly have happened to you because you're not upset by it … what's happened is so awful that they ought to be suffering in some way, and therefore if they're not, then perhaps that's a lie. (Participant 7)

Participants’ recognition of mental health problems in their clients was supported by training and experience. Participants who had received training on mental health issues identified this as being very helpful for their decision-making.

They [the Medical Foundation for the Care of Victims of Torture; now Freedom from Torture] come here and do training for us about identifying different mental health presentations so I suppose we are a bit more aware and know how to respond and to detect whether there is a problem basically. (Participant 2)

Experience of seeing many clients diagnosed with PTSD and personal experience of distress were also identified by some as being helpful.

I think I could make some judgement about whether someone is suffering from PTSD, largely because I see so many poor souls. (Participant 4)

More personal characteristics were also identified by participants as influencing their decision-making. Some participants identified the importance of using their instinct, getting a feeling that “something is not quite right” with a person and this being seen as a potential indicator of mental health problems. Participants who spoke of this feeling found it quite difficult to identify what exactly led them to it.

You get a gut instinct … I don't think it's something that you can learn, not something that you can have a chart and have a tick box, I think that's impossible. (Participant 10)

Personal characteristics included drawing on individual experience.

Perhaps you do use a bit of your individual experience … I can say from my background, I know what it's like to have lost somebody, so I have been through all that grieving process. (Participant 5)

Identifying a client as possibly having mental health problems also seemed to be linked to the legal representatives’ recognition of a level of distress in the client that they did not feel able to cope with. When speaking about clients they had referred for reports, participants often identified that they had felt great concern for these clients and that they could not meet the needs of the client themselves.

I feel scared as a lawyer, that I've taken these things out of the Pandora's box, but I don't have any method of counselling or comforting or telling the person how to deal with it … The most extreme was that I was actually frightened that my client on leaving the office might just go straight under a passing lorry, because she felt literally that there was no point in bothering to carry on. (Participant 4)

And it was the wife that brought that to our attention, saying that my husband is talking about suicide, and I've had to make a referral to a specialist, because I can't deal with it. (Participant 3)

Others considered the needs of the client and expressed a desire to assist clients in accessing appropriate help, referring for treatment both to get a report to strengthen the case and to help the client more directly.

… generally I'm trying to refer them for their own benefit as well. They may need counselling, or medication. (Participant 11)

### Barriers to obtaining reports

Participants’ decisions to refer for a report did not always result in a report being obtained or used as part of the case. Several barriers were identified which could halt this process. Some barriers occurred within the legal system, including difficulties in obtaining funding or negotiating adjournments. Other barriers occurred in the medical system, including long waiting times, high fees and difficulties in finding a suitable expert. It appeared that the failure to get a report could result from the clash between these two systems; for example, one treatment provider wanted 6 months to prepare a treatment report, whereas the courts refused to grant an adjournment for this.

A further barrier that was identified was the client's attitude to the referral.

There are occasions when it is more difficult because the client may feel that there's something detrimental about being told well we want you to be seen by a doctor, you know who's a psychiatrist, and they think ‘oh you think I'm mad’. (Participant 7)

## Discussion

This is the first study, to our knowledge, to systematically examine clinical judgments made about asylum seekers by immigration lawyers – non-clinical decision makers – in the asylum process, using established qualitative research methods.

One of the most striking findings was that legal representatives in this study were making decisions about the presence of mental health problems in their clients based on their lay knowledge of mental illness and their own responses to the presentation of the client. The legal representatives interviewed for our study were highly experienced and motivated lawyers who see it as part of their role to seek extra training and knowledge about the mental health issues that might affect their clients. Nonetheless, legal representatives do not generally come from a background of clinical training and experience.

### Recognising post-traumatic stress

Participants cited professional and personal experience, training and instinct as helping them to make these quasi-diagnoses. However, while some participants identified specific – and correct – questions regarding nightmares and flashbacks (which are “re-experiencing” symptoms of PTSD), questions about other symptoms, such as avoidance and hyperarousal symptoms were less used. Other participants relied more on the level of distress displayed by clients or a “gut” instinct that something was amiss.

This suggests that representatives and decision makers may rely on lay understandings of distress that do not necessarily fit with all possible presentations of psychological disorder. This was also found in a study of psychological issues in refugee applicants in Australia (Hunter et al., 2010). Seven of the applicants followed in the study displayed emotional detachment at their hearing. None of the decision makers involved made any comment about the dissociative presentation of the applicants, despite psychology reports drawing attention to the condition and explaining its potential impact on demeanour. All seven applicants were refused refugee status on credibility grounds, suggesting that presentations of PTSD which are less well-understood by lay decision makers may pass unrecognised.

A recent UK study explored these issues, using an analogue design. An actor was instructed to present an asylum story in four different ways. Firstly, with outward signs of PTSD (constructed from clinical literature and expert consultation); secondly, with known cues indicating deception (from literature, e.g., Vrij, 2008); thirdly, with cues indicating both PTSD *and* deception, and, finally, with none of these outward signs. Students, instructed in asylum decision-making, then made credibility judgments about each presentation. The presentation deemed most credible was the PTSD-alone picture, which on the face of it is an encouraging finding for those in the asylum system, struggling to be recognised as psychologically vulnerable. However, further qualitative exploration suggested a role played by “emotional congruence”, a construct described by Kaufmann, Drevland, Wessel, Overskeid, and Magnussen (2003), which showed that the perceived credibility of a simulated rape victim's statement increased if the victim showed despair. This expectation of “appropriate distress” is also seen in the present study, where there was an expectation that rape, trafficking or torture “should” result in discernible psychological effects.

If signs of distress are not understood by lay assessors and clients are not vocal about their needs, they may be less likely to get help or be assessed as psychologically vulnerable. This issue is also present within the health care system, for asylum seekers, refugees (Ward & Palmer, 2005) and other client groups (e.g., depressed older adults; Age Concern, 2008).

### Circular argument

This question of making judgments based on how someone “should” present if they have experienced persecution raises other important issues, predicated as it is on an assumption that past persecution has taken place. This risks mixing the perceived “facts of the case” with judgments about the need for procedural adaptations for vulnerable applicants. Current guidelines for excluding people from the UK DFT require that there be “independent evidence” of torture (Home Office, 2012b). Putting aside the claimant's difficulty in obtaining such evidence at an early stage in their claim, this introduces a fact-finding element into a decision which is primarily intended for the protection of potentially vulnerable individuals. If someone is more than 24 weeks pregnant, or acutely ill, then putting them in a detention centre would be unduly harsh and accepting such exclusion criteria should be possible without making any concessions concerning the merits of their claim. However, to accept a history of torture (via the “independent evidence”) is already to begin to accept an account of persecution which could support a claim for protection and this may lead to a reluctance on the part of decision makers to make judgments on psychological vulnerability in isolation.^3^

So the argument expressed by some participants in our study that “if rape/torture/persecution happened to someone then they would have psychological difficulties” is prey to the counter-argument “we don't believe it did happen, so we don't accept that the difficulties are genuine”, as frequently seen in responses to medico-legal reports themselves (Good, 2004, pp. 203–204).

It also runs counter to the clinical literature. Johnson and Thompson's survey of studies of civilian adult survivors of war trauma and torture found that the prevalence of PTSD in non-clinical populations can be as low as 12–26% (Johnson & Thompson, 2008). A study of those who have suffered sexual assault, found a current prevalence of 32% (community sample; Resnick, Kilpatrick, Dansky, Saunders & Best, 1993). This leaves a significant number of people who, whilst they have experienced significant distress, loss or trauma, may not be experiencing any of the particular psychological difficulties that would be identified as indicating vulnerability.

### Role of depression

The participants’ perception of depression also seemed to be at odds with what is known from clinical research. Depression seemed to be viewed as not severe enough to warrant bringing to the court via a medico-legal report. However, research has shown that depression does have significant effects on memory, leading to a bias towards negative events and, perhaps more relevant here, a tendency towards overly general memory (Williams, Watts, MacLeod & Mathews, 1997), which could lead to an account lacking detail and thus being judged as lacking credibility. Thus, it seems that a diagnosis of depression could potentially be submitted in court as a possible explanation of failure to recall details; evidence which may not be currently recognised by legal representatives.

What was recognised by our sample of immigration lawyers was stated suicidal ideation. However, the judgement of actual suicidal risk is one that, in clinical services, is subject to much research-based guidance and training^4^ and it is not clear from our findings how much of such training would be available to these lawyers or indeed other actors in this area, such as state decision makers.

### Subjective reasons for referring

Representatives’ own comfort levels informing their decision to call in expert assistance with a case adds a subjective element to their judgments. It also raises the question of what else is contributing to those comfort levels. A Canadian study showed how refugee tribunal members employed “direct avoidance, denial and trivialization of extreme events” as protection against traumatic material, [or ‘but’] to the detriment of their decision-making (Rousseau, Crepeau, Foxen & Houle, 2002). Studies in the United Kingdom have shown burnout and vicarious traumatisation in interpreters (Johnson, 2009) and volunteer supporters (Guhan & Liebling-Kalifani, 2011) working with refugees and asylum seekers, and a qualitative study of immigration lawyers identified the “emotional labour” necessary to manage conflicting roles of empathic advocate with objective fact-finder (Westaby, 2010). Further quantitative studies are needed to establish the role of vicarious traumatisation and its effect on decision-making throughout the asylum process.

## Limitations

There are limitations to this study. The first lies in the small and potentially unrepresentative sample of participants: only 12 of over 700 potential participants came forward to participate. Qualitative research typically relies on small samples, and a low response rate is perhaps to be expected when recruiting via e-mail; however, in terms of generalisability, this limits the conclusions one can draw. It is highly likely that those who participated did so because they had a particular interest in the use of psychological medico-legal reports in asylum cases or knowledge of mental health issues. Thus, the sample for this study may be an unusually well informed and motivated group of legal representatives. How a similar study of a sample of decision makers would compare remains an empirical question that could usefully be addressed in a future study.

The interviews may have produced biased data due to a perception of the interviewer as someone with expert (mental health) knowledge (Yang & Yu, 2008) and the influence of social desirability on participants’ responses to questions. However, an effort was made to minimise the impact of this by stressing at the start of the interview that the interviewer had no expertise in this area and was taking a “naïve” approach. In addition, participants were asked to talk through specific cases, which may have helped to minimise global, positive, general accounts.

This study did not explore the accuracy of legal representatives’ recognition of mental health problems. Given the poor rate of recognition of mental health issues, particularly PTSD, amongst even medically trained staff, future research could helpfully compare the clinical judgements made by legal representatives, border agency decision makers or other lay actors in the asylum process to those made by mental health professionals, experienced and qualified in making psychiatric diagnoses.

## Conclusion

This study has started the work of shedding light on how clinical assessments of asylum seekers’ mental health are made by non-clinical personnel in the asylum process. Freedom from Torture (formerly the Medical Foundation for the Care of Victims of Torture) suggests that identification of psychologically vulnerable asylum seekers should occur at the earliest possible stage of any asylum procedure and recommends the medical assessment – by a well-trained clinician, focusing on health and well-being rather than their asylum claim – of all asylum seekers upon arrival (Jones, 2007). Others have proposed screening checklists, to be used by lay immigration staff, for example, the PROTECT project,^5^ recently launched in Belgium, which is based on clinical assessment research and is in line with the Istanbul Protocol (United Nations, 2004). However, no system has as yet been uncontroversially accepted.

This study of what we have termed “clinical judgments” as made by non-clinically trained personnel may appear to emanate from professional protectionism. This is not our intention. It may be that training of state, legal and judicial staff can provide a route to the effective recognition of the people most in need of help to navigate their way through the asylum system and provide appropriate evidence to support their claims. However, the results of this study, taken together with existing clinical literature, suggest that this would have to involve more than a checklist of observable symptoms. A valid and reliable judgment of vulnerability requires the decision maker to step outside the discourse of credibility assessment, to exercise neutrality as to causality, to take a critical stance to their own socio-economic, cultural and historic background and associated views and stereotypes, to draw on a thorough understanding of clinical literature and to recognise unusual as well as the more common presentations of psychological distress.

## Footnotes

1 Barring exclusions, for example, those convicted of serious non-political crimes in other countries, perpetrators of crimes against humanity, etc. (see, e.g., Good, 2004).

## Footnotes

2 Procedure Directive http://eur-lex.europa.eu/LexUriServ/LexUriServ.do?uri=OJ:L:2005:326:0013:0034:EN:PDF introduces a minimum framework in the community on procedures for granting and withdrawing refugee status.

## Footnotes

3 Indeed, in the United Kingdom case workers are required NOT to separate out any aspect of the “evidence” but to review all evidence in the round (see UK Border Agency, 2011).

## Footnotes

4 For example, see http://bestpractice.bmj.com/best-practice/monograph/1016/diagnosis/step-by-step.html.

## Footnotes

5 see http://www.pharos.nl/information-in-english/protect (last accessed October 8th, 2012).
